# Supplementary material for: Preclinical Evaluation of the Association of the Cyclin-Dependent Kinase 4/6 Inhibitor, Ribociclib, and Cetuximab in Squamous Cell Carcinoma of the Head and Neck
Source: Cancers (Basel). 2021 Mar 12;13(6):1251. doi: 10.3390/cancers13061251 (PMC7998503; doi:10.3390/cancers13061251)

Western blots were revealed with Spectramax and analyzed with ImageJ (excepted for pSTAT3Ser727):

Spectramax:

Rb total:
(#9303)

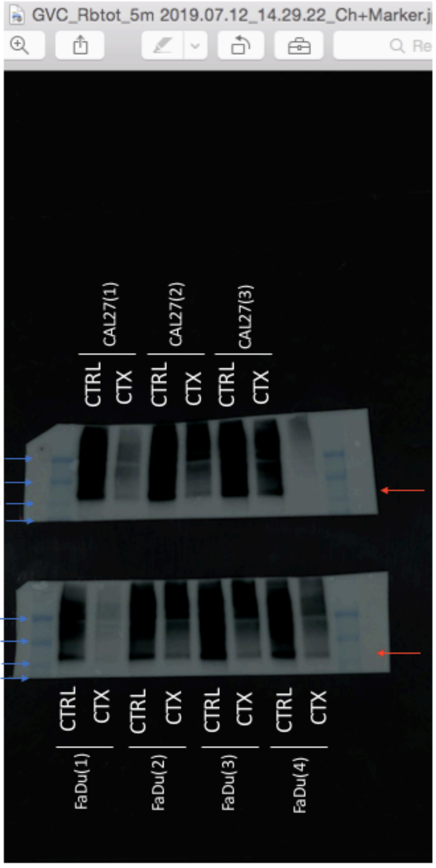

ImageJ:

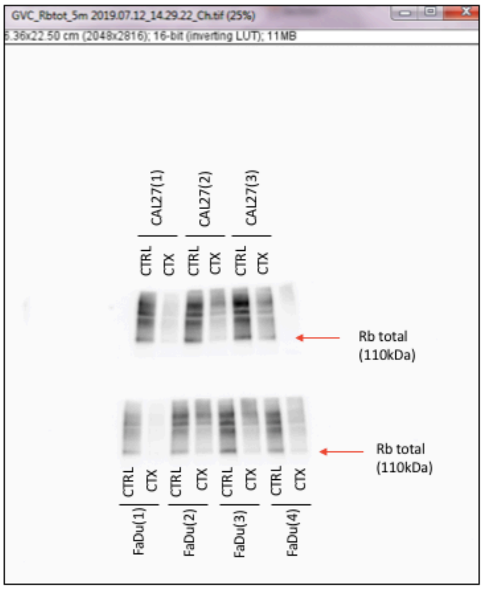

Spectramax:

pRb Ser795:
(#9301)

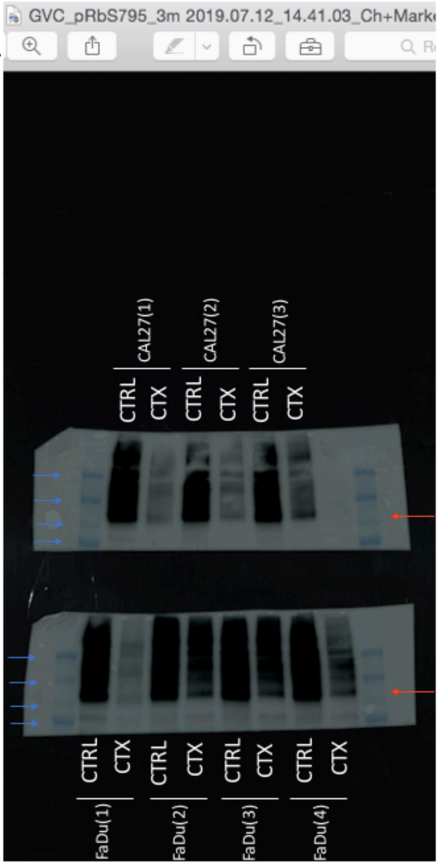

ImageJ:

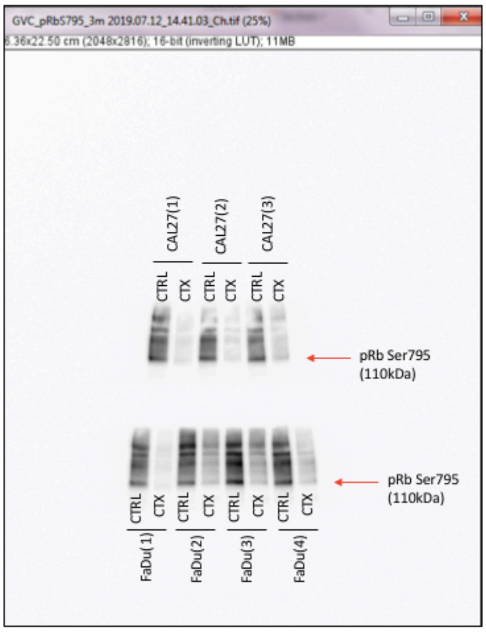

Spectramax:

P107:
(sc-318)

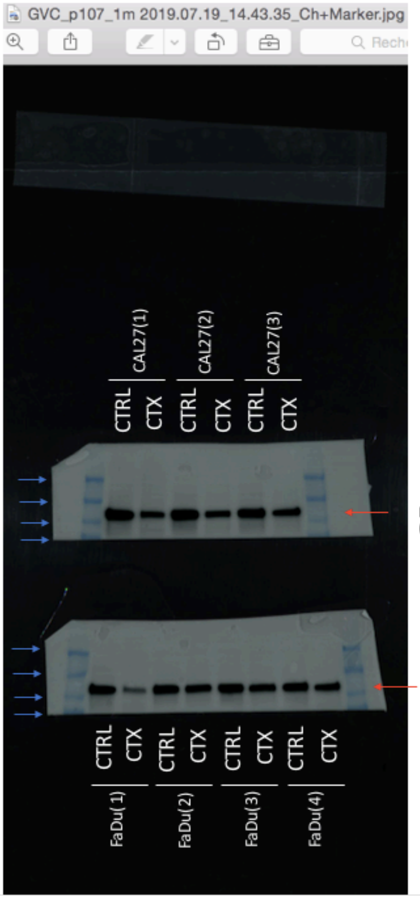

ImageJ:

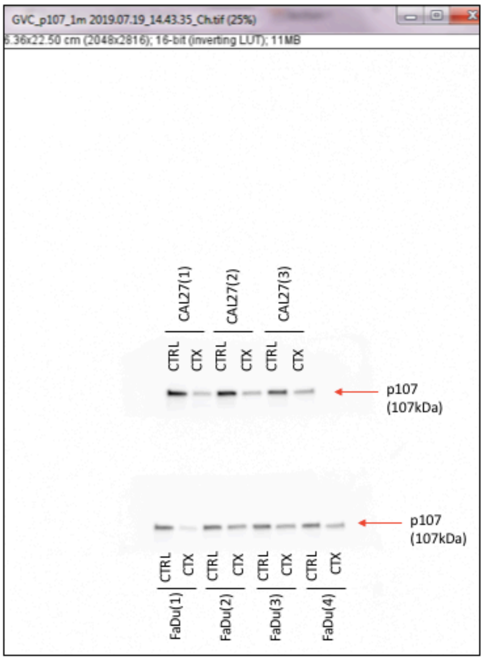

Spectramax:

P130:
(sc-317)

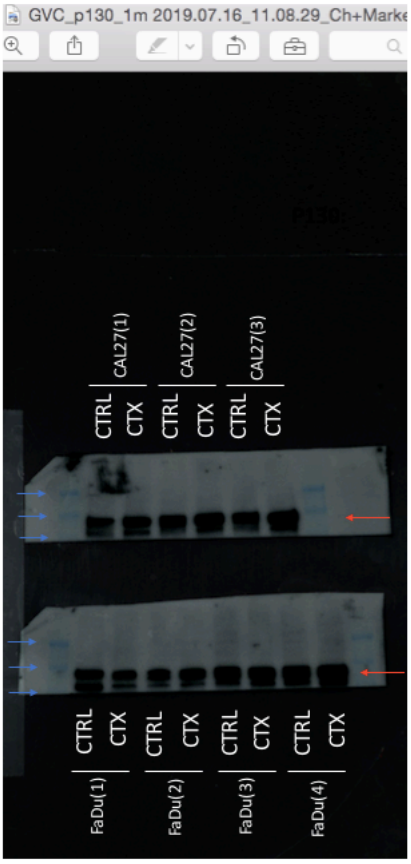

ImageJ:

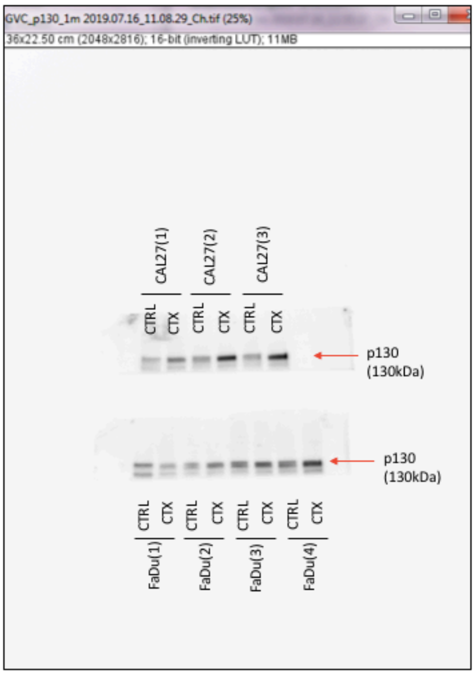

Spectramax:

CDK2:
(sc-6248)

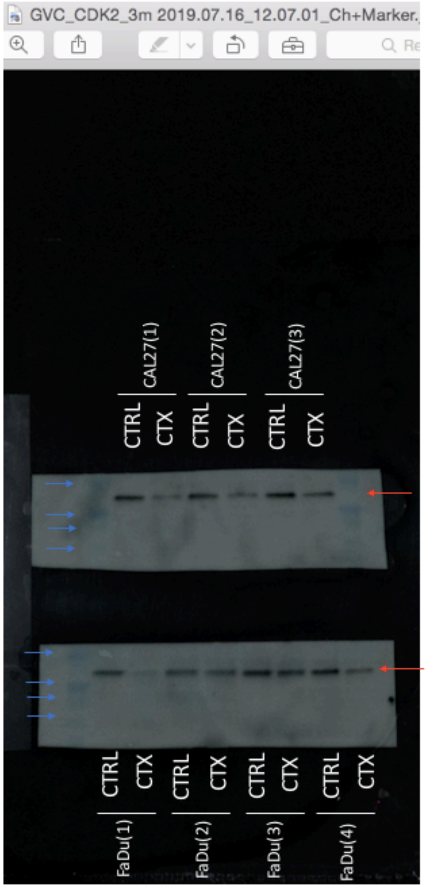

ImageJ:

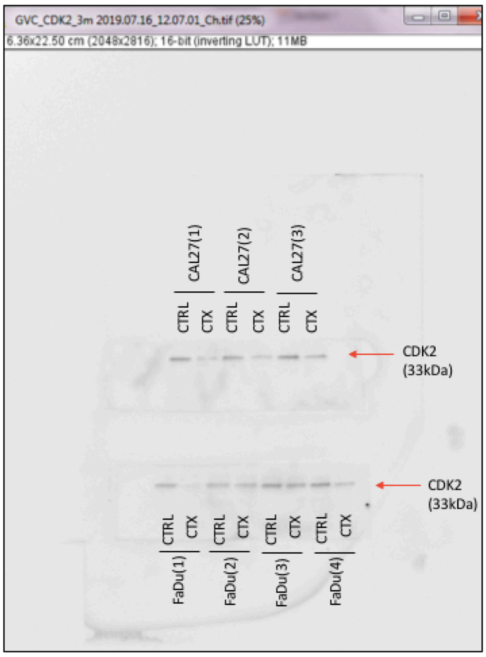

Spectramax:

CDK4:
(#12790)

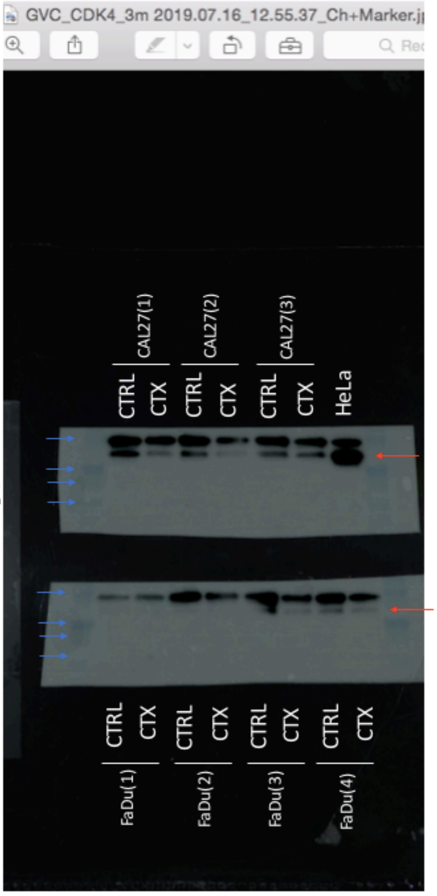

ImageJ:

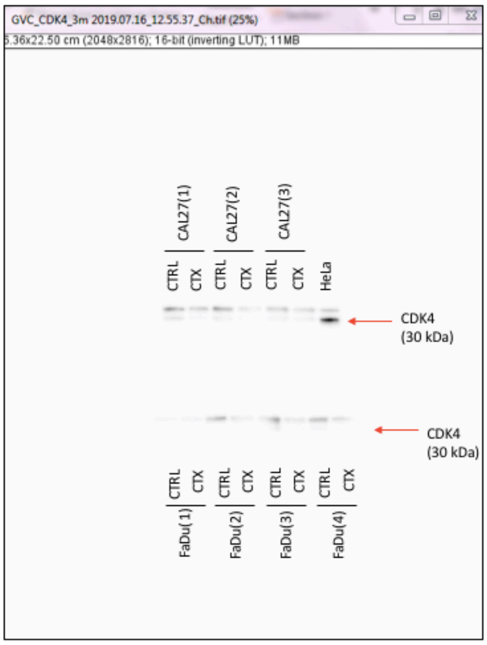

Spectramax:

CDK6:
(#3136)

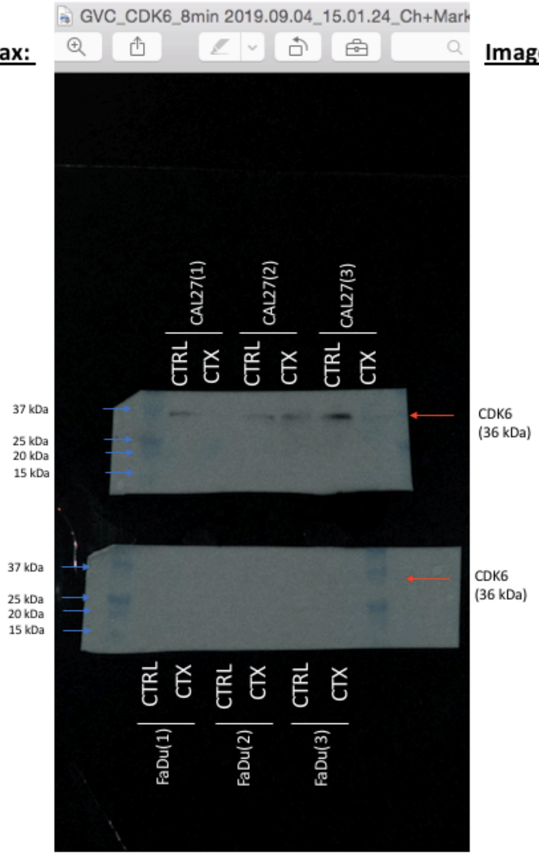

ImageJ:

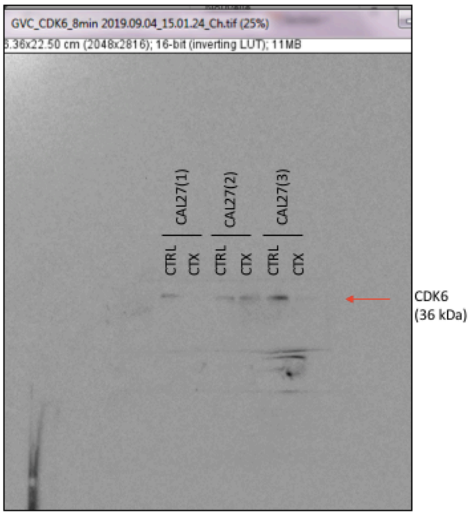

Spectramax:

Cyclin A2:
(#4656)

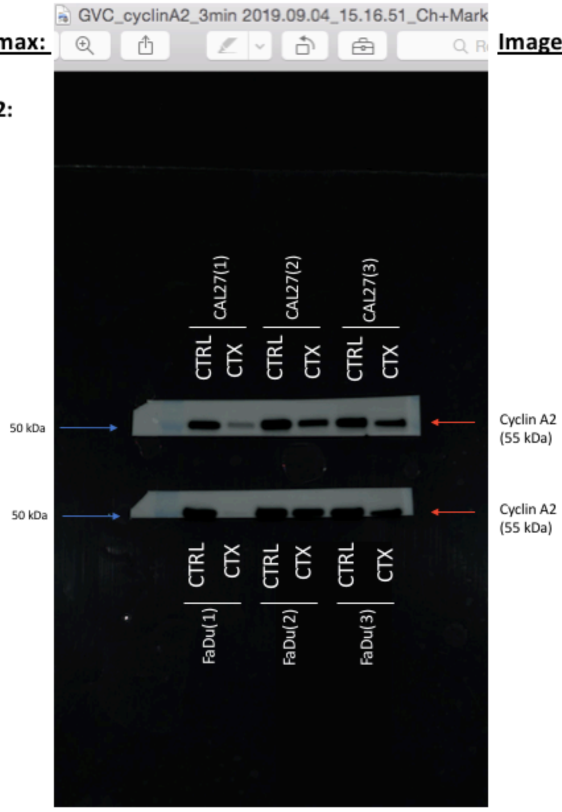

ImageJ:

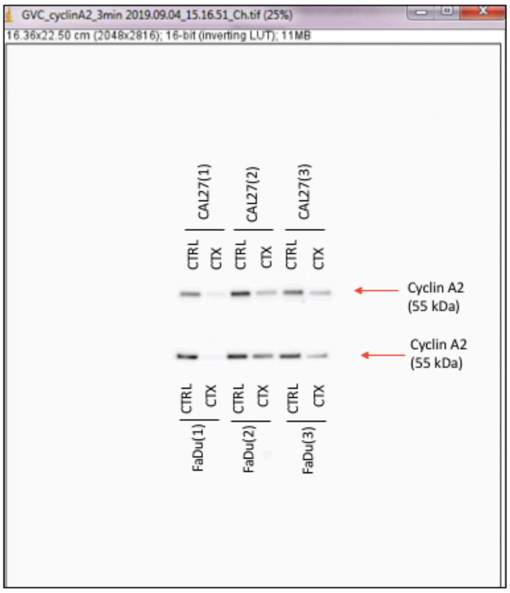

Spectramax:

ImageJ:

Cyclin D1:
(#2922)

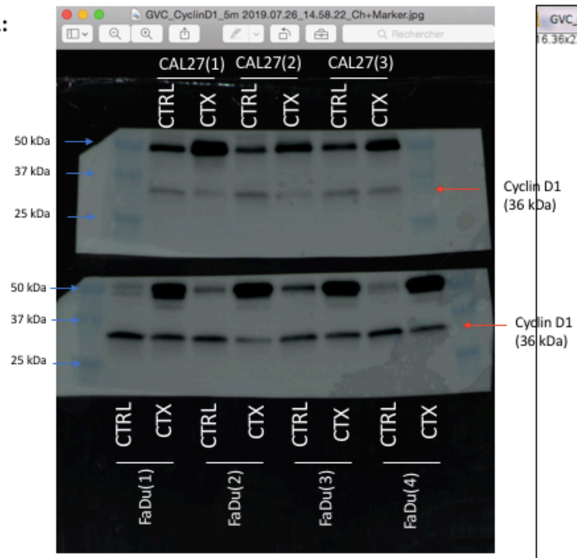

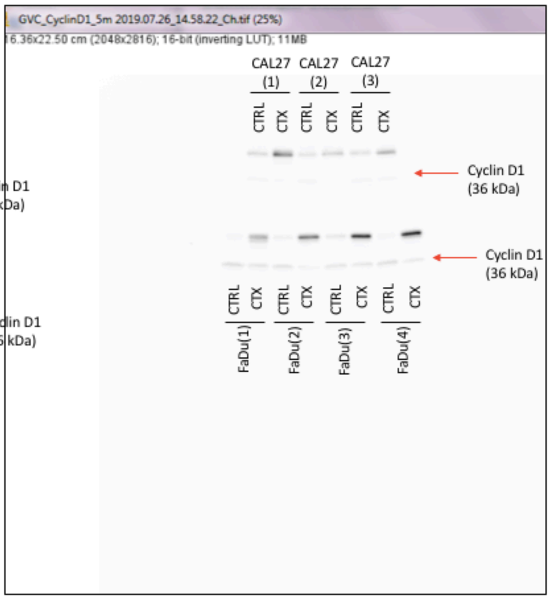

Spectramax:

ImageJ:

Cyclin E1:
(#4129)

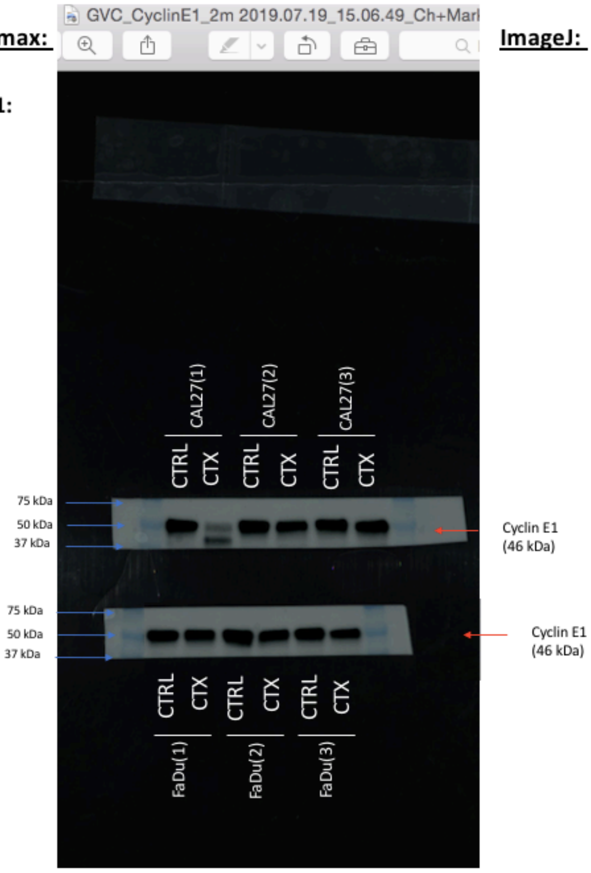

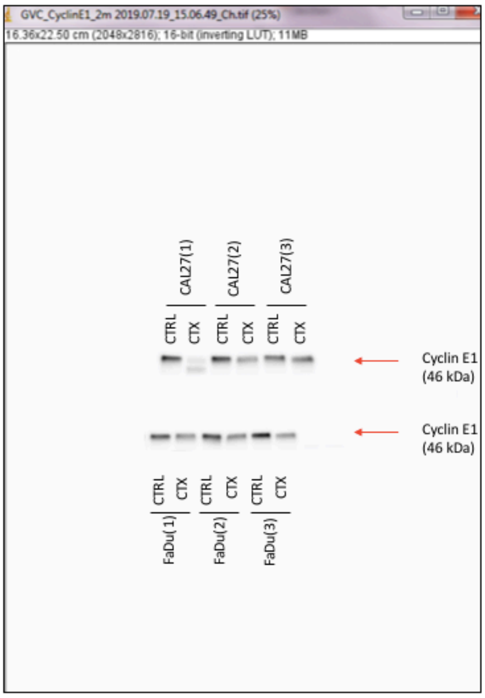

Spectramax:

E2F1:
(#3742)

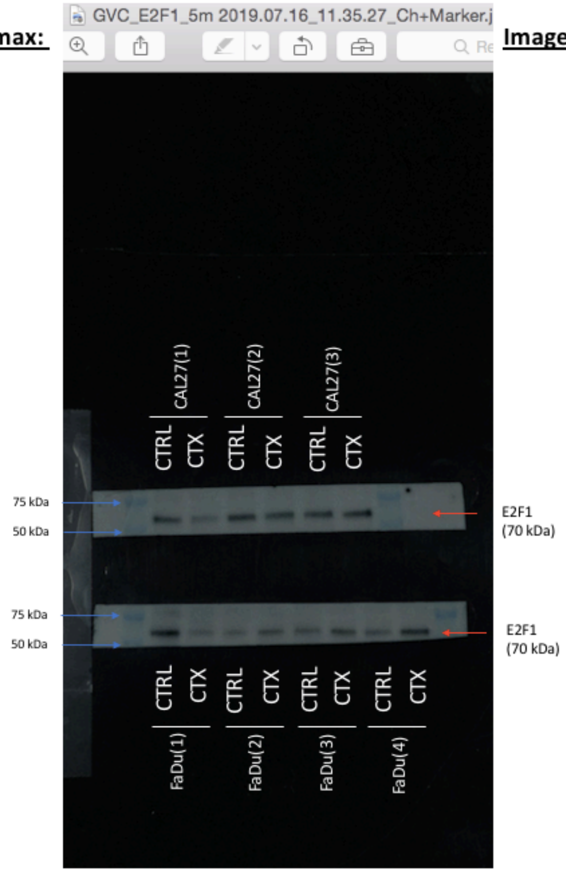

ImageJ:

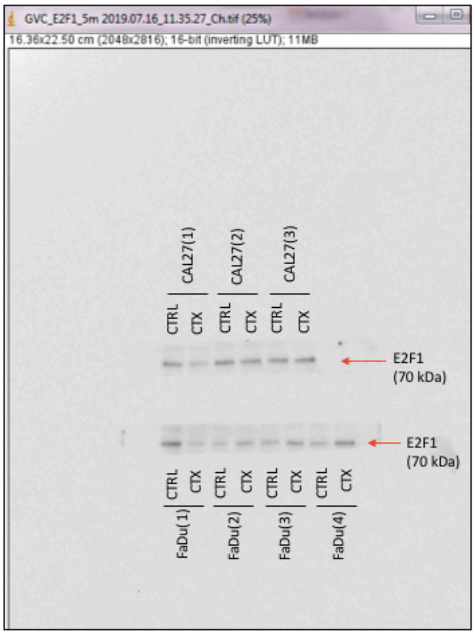

Spectramax:

c-myc:
(5605)

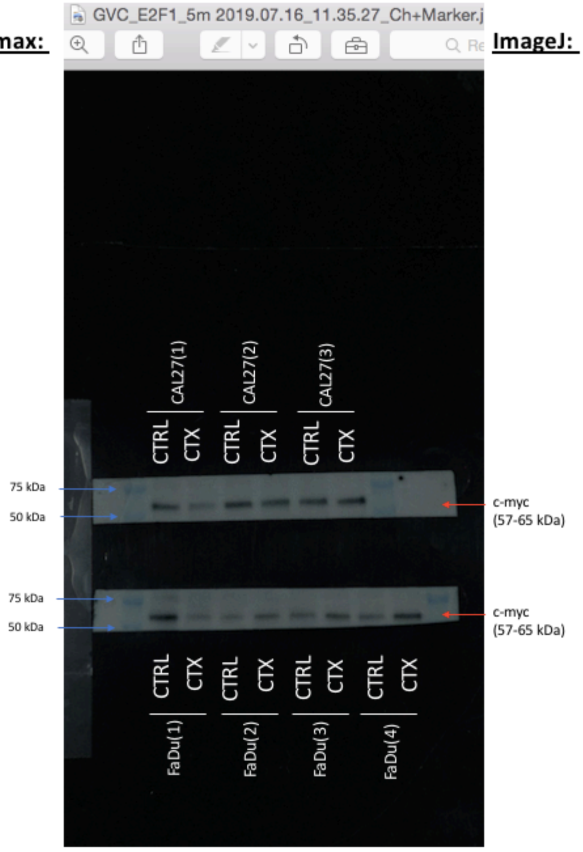

ImageJ:

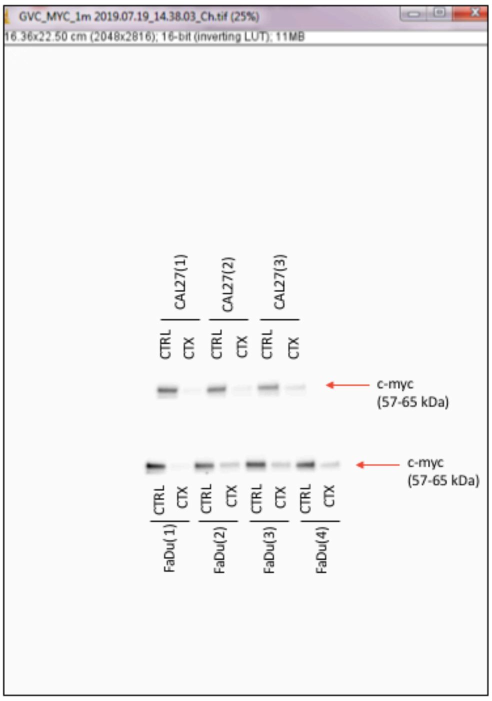

Spectramax:

FOXM1:
(sc-500)

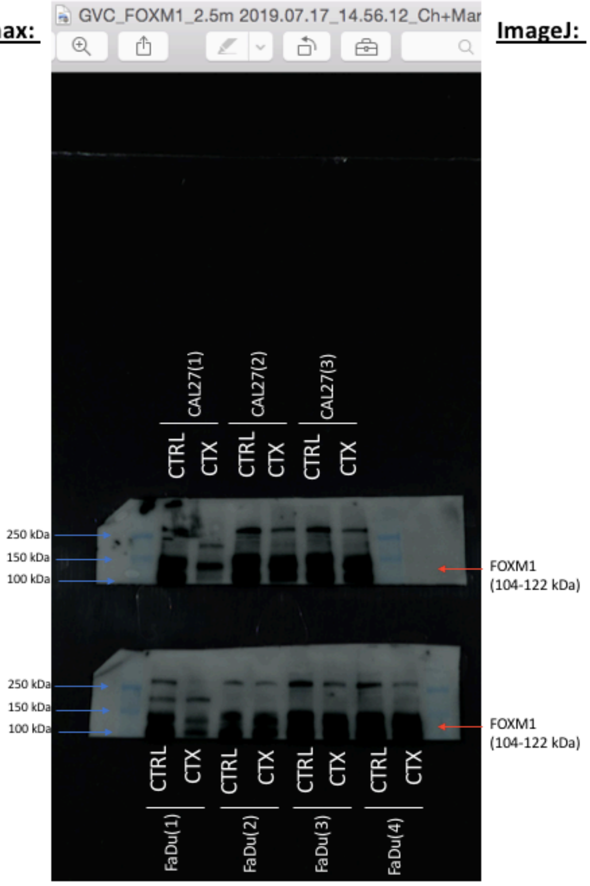

ImageJ:

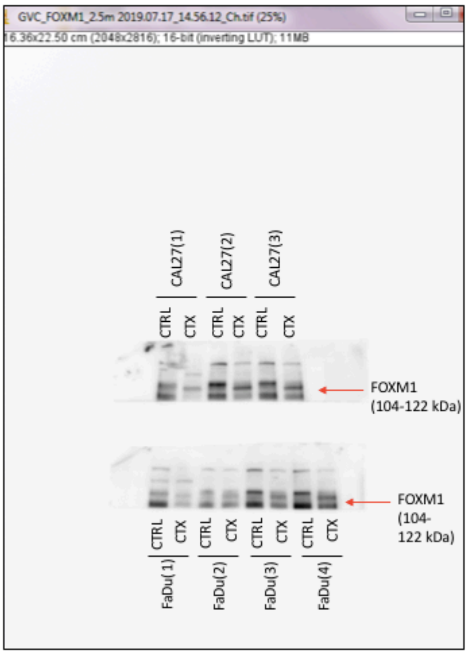

Spectramax:

GAPDH:
(G9545)

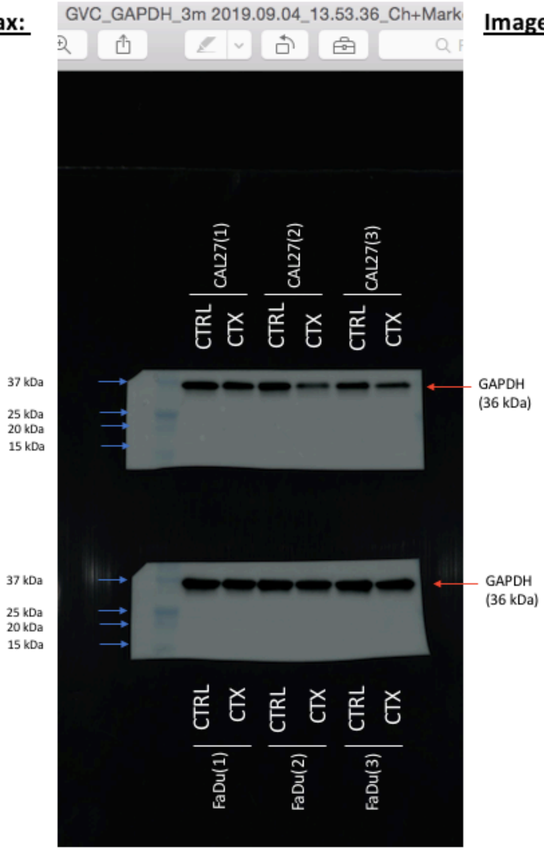

ImageJ:

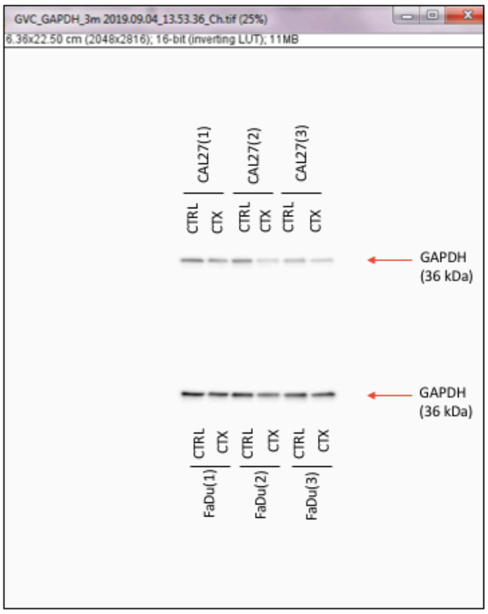

Spectramax:

EGFR total: (#4267)

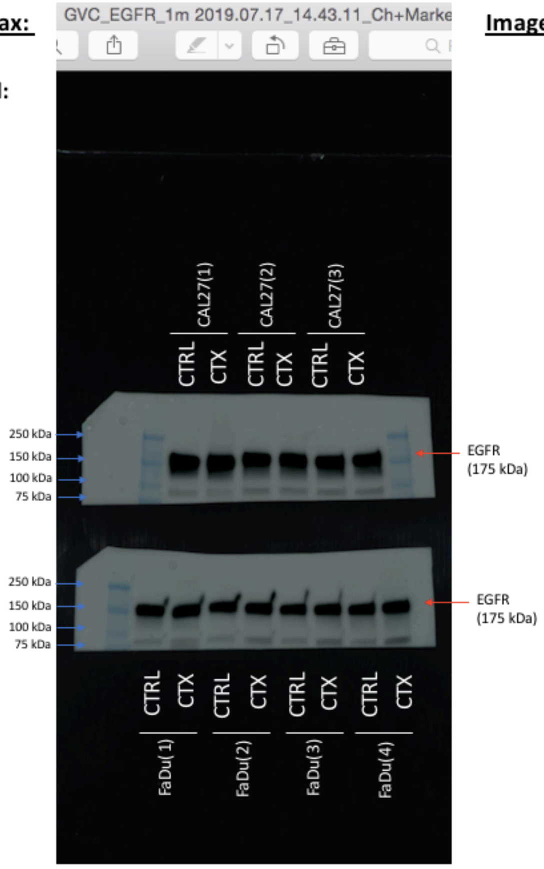

ImageJ:

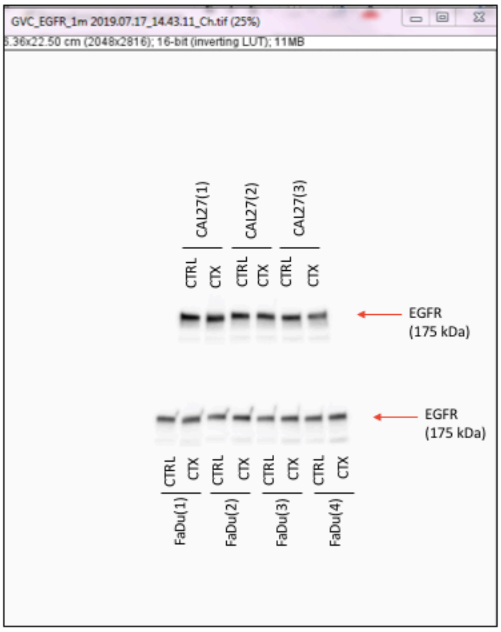

Spectramax:

pEGFR Tyr1068: (#3777)

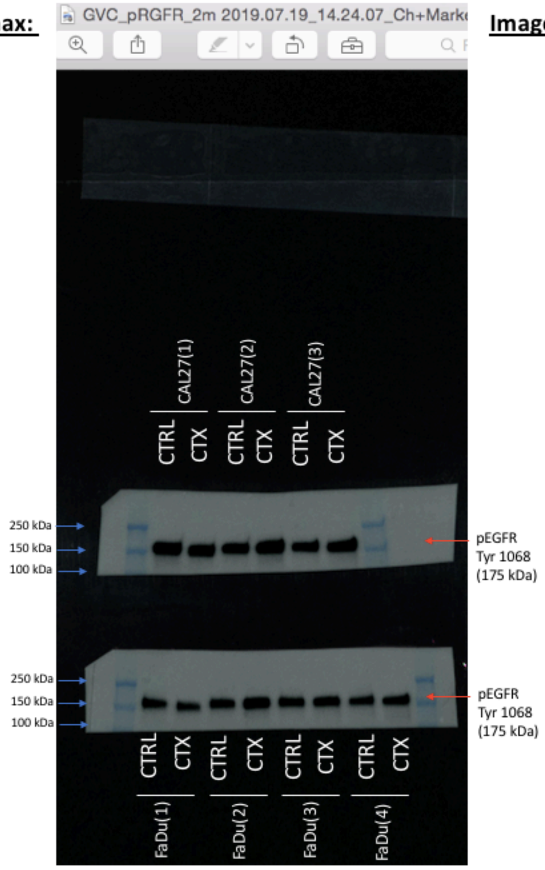

ImageJ:

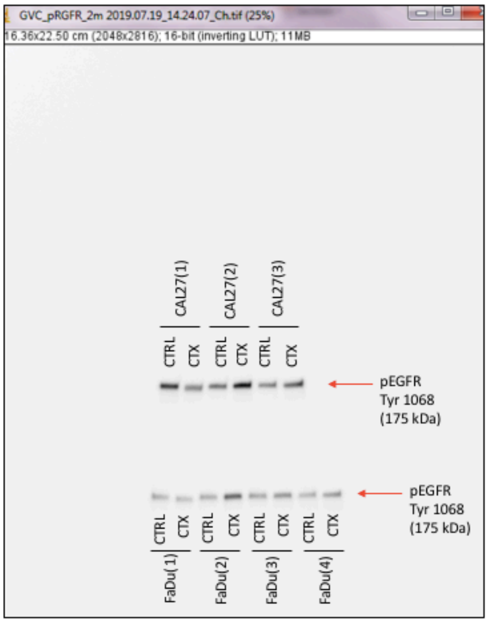

Spectramax:

Akt total:
(#4691)

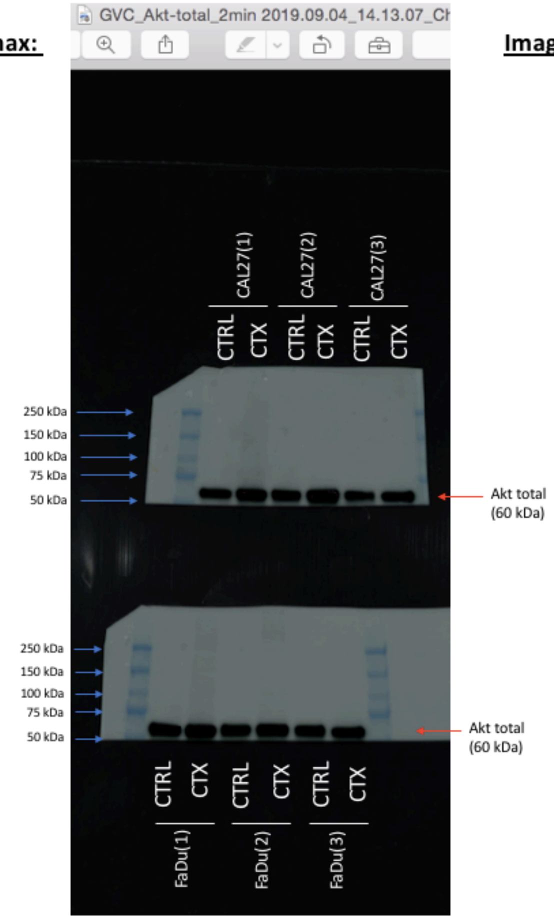

ImageJ:

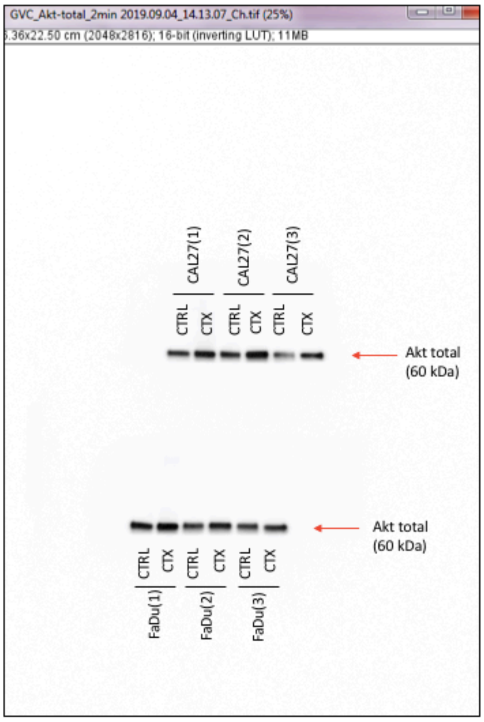

Spectramax:

pAkt
Ser473:
(#4060)

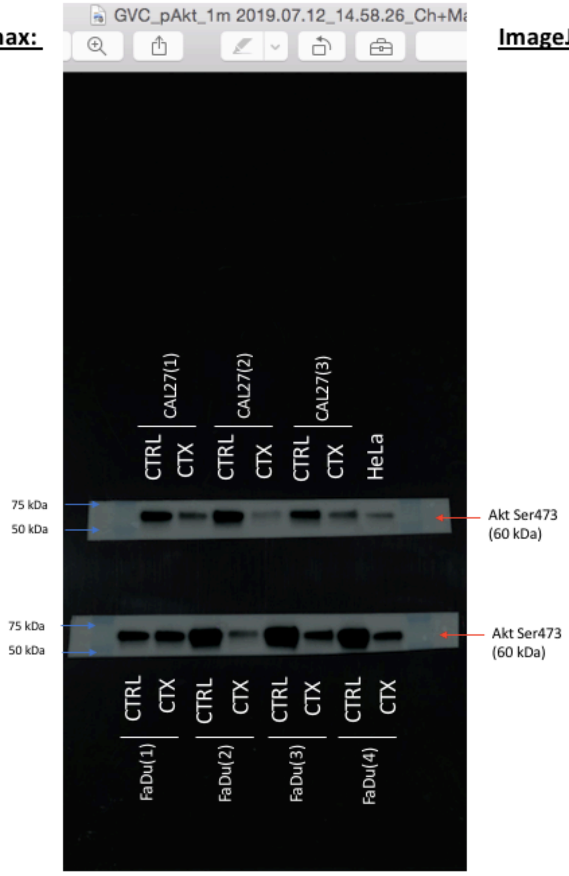

ImageJ:

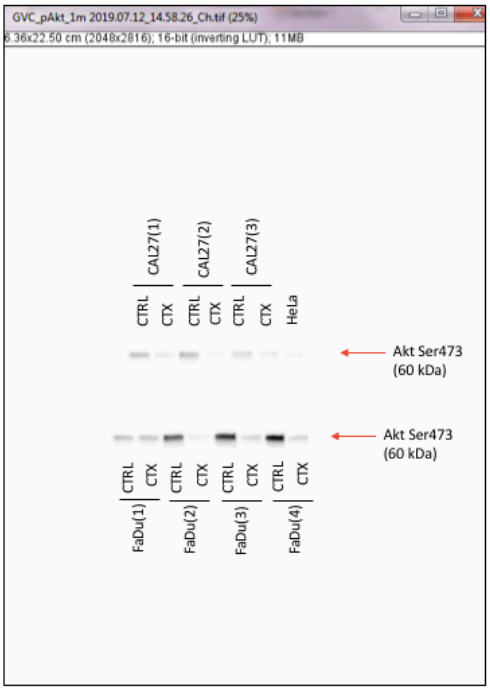

Spectramax:

ERK1/2 total: (#9102)

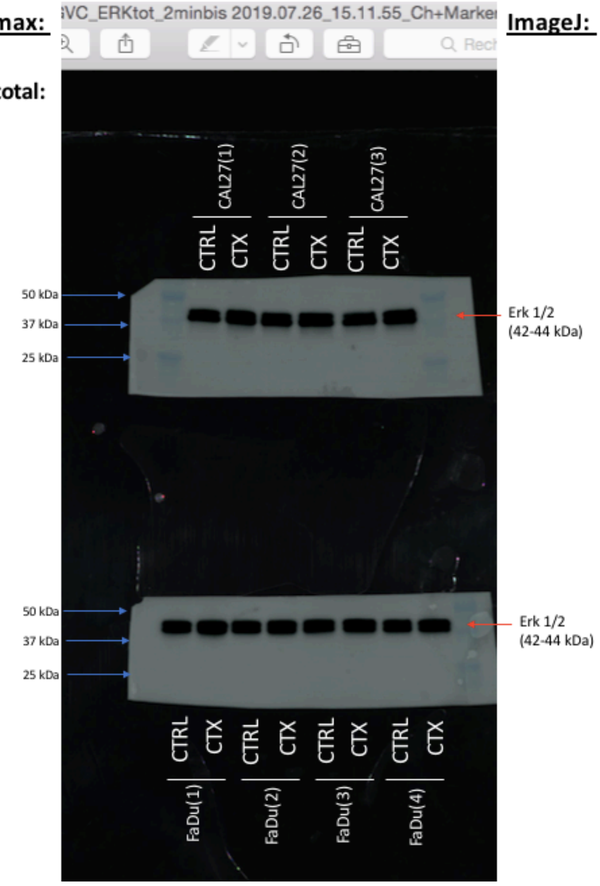

ImageJ:

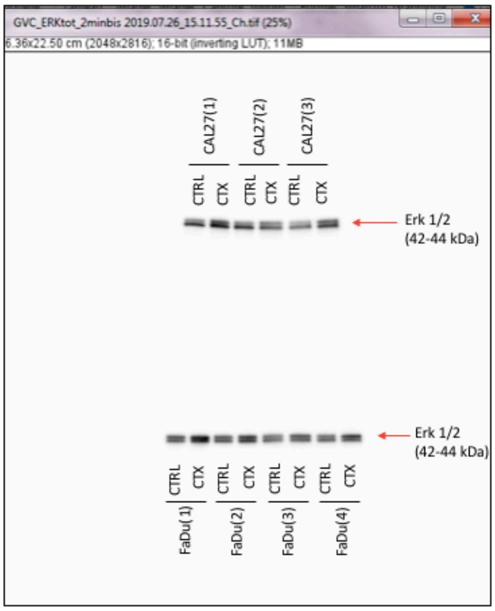

Spectramax:

pErk1/2 Thr202/Tyr204: (#4370)

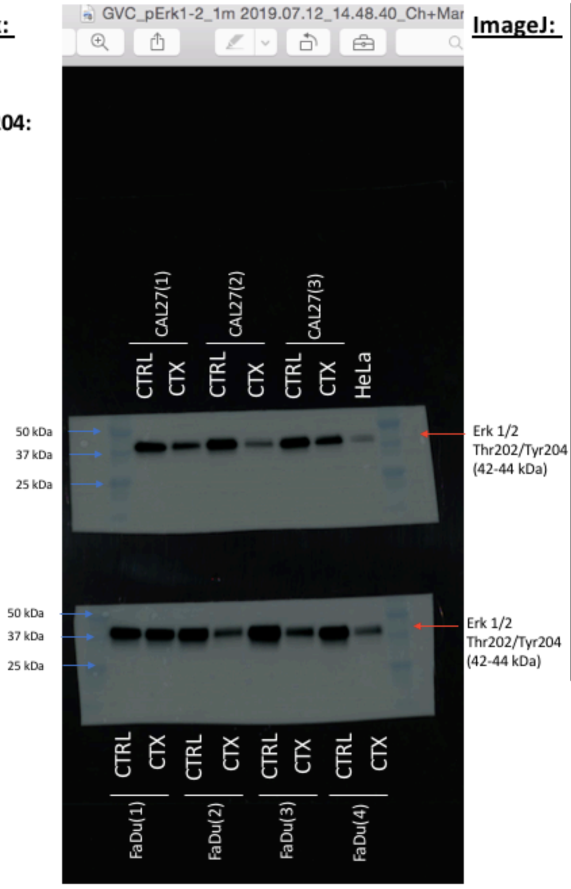

ImageJ:

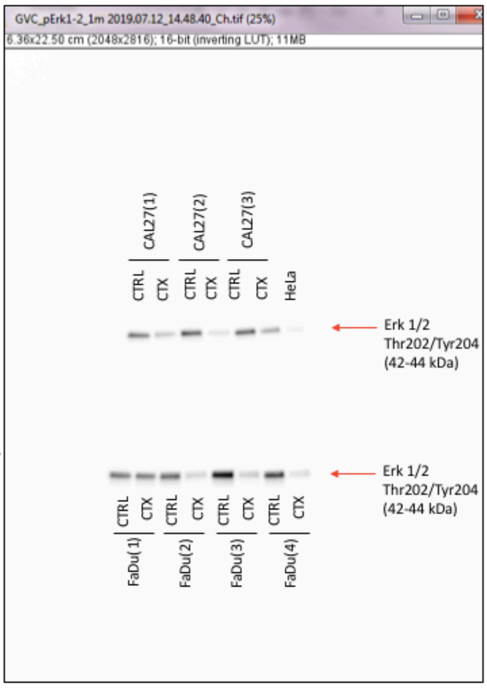

Spectramax:

STAT3 total: (#9139)

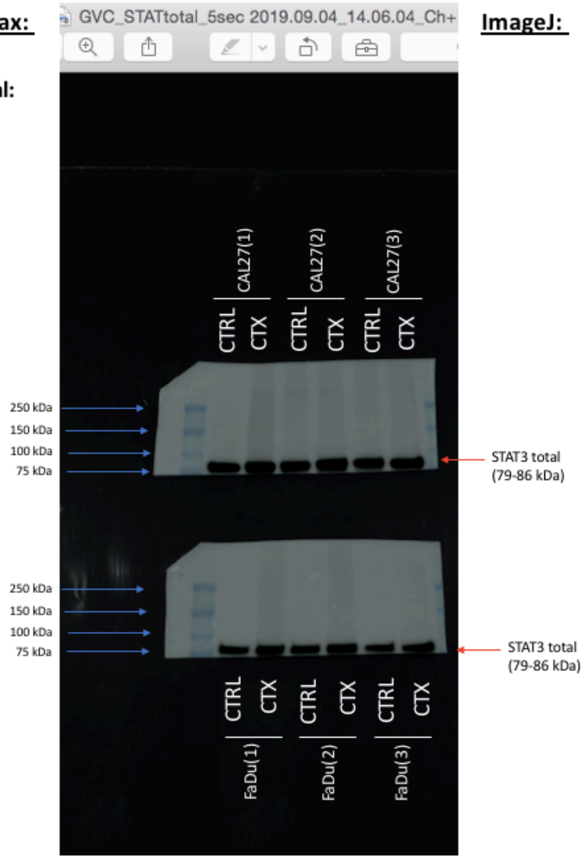

ImageJ:

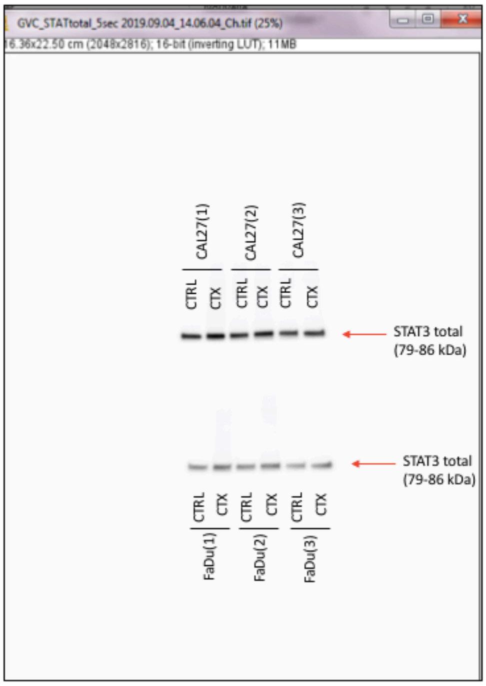

Gel

pSTAT3 Ser727: (#9136)

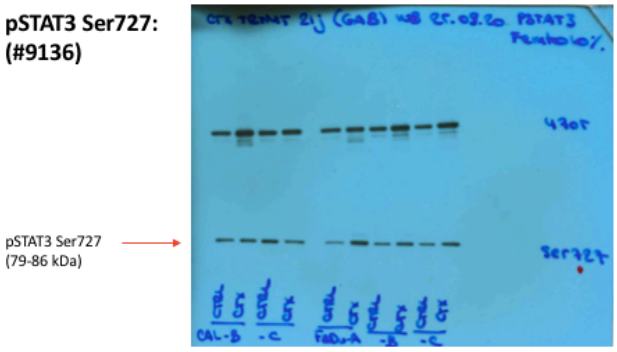

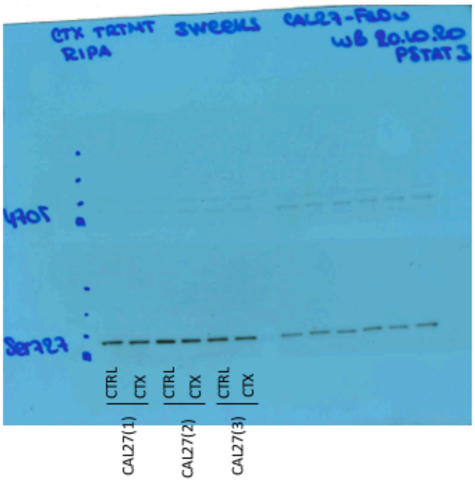

Spectramax:

pSTAT3 Tyr705: (#9145)

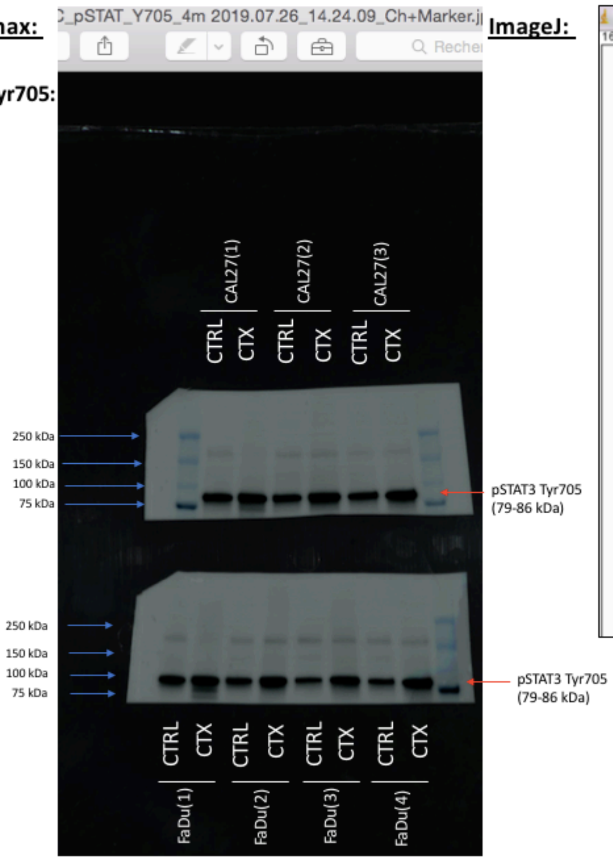

ImageJ:

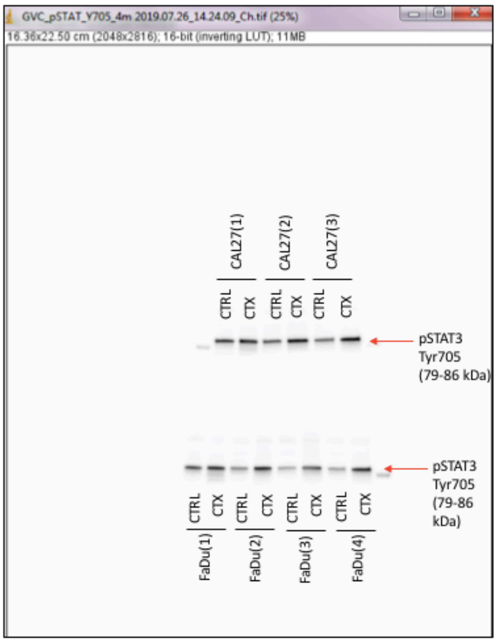

Spectramax:

MDM2 total: (#86934)

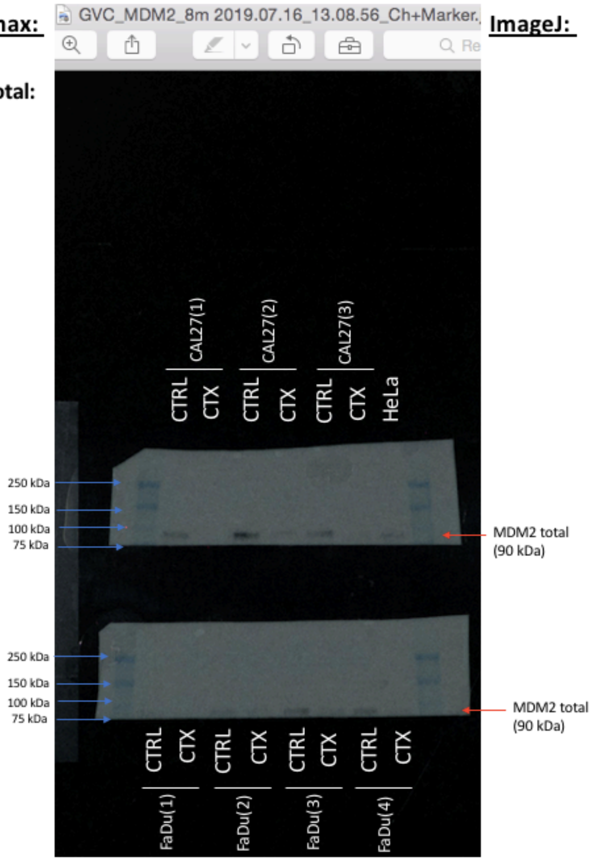

ImageJ:

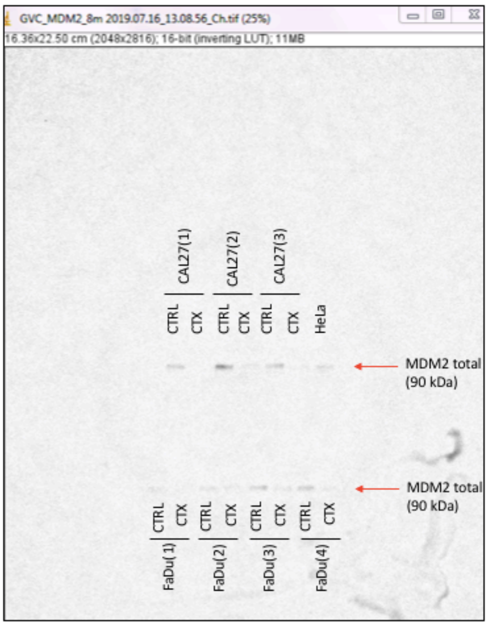

Spectramax:

pMDM2 Ser166:
(#3521)

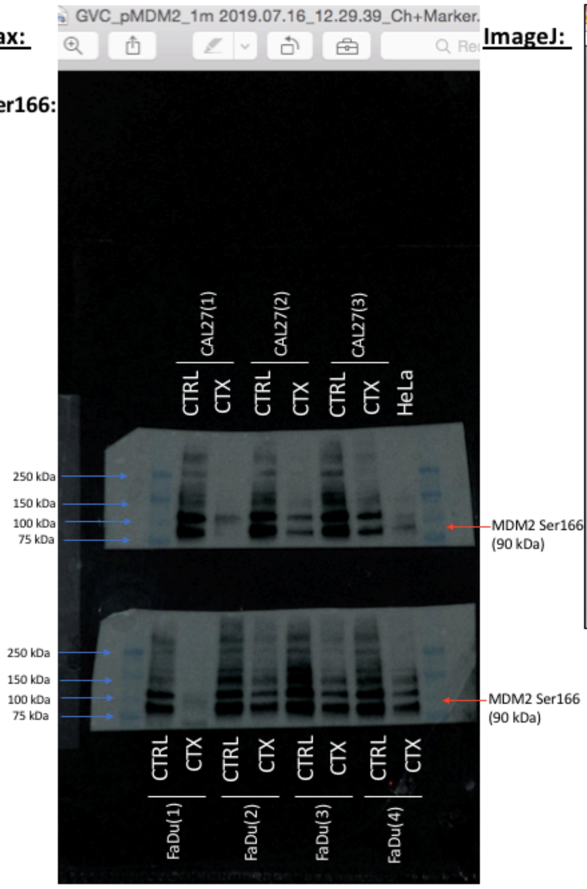

ImageJ:

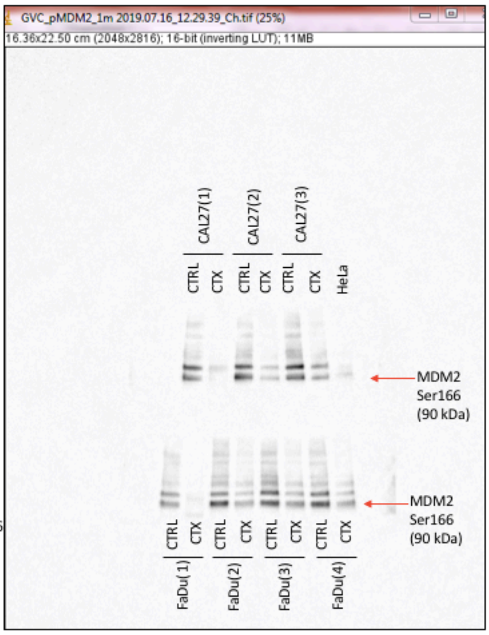

Spectramax:

GAPDH:
(G9545)

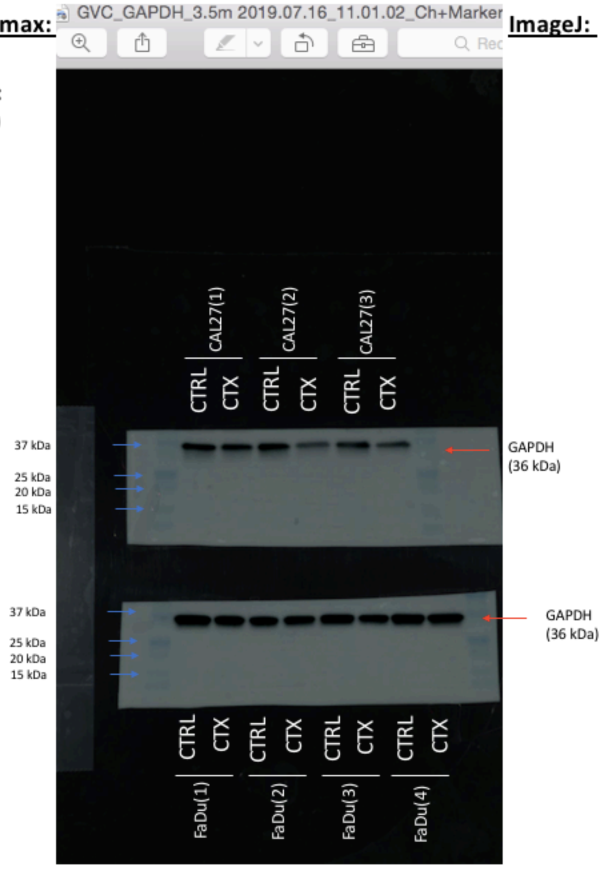

ImageJ:

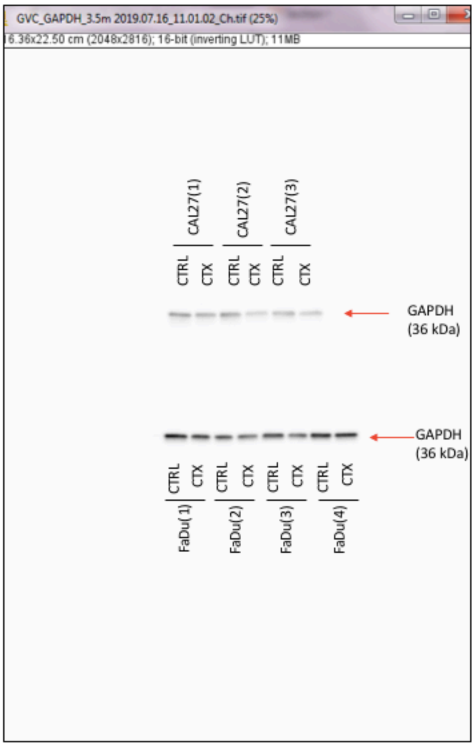

Supplement: Supplementary file 1 [file cancers-13-01251-s001.zip › cancers-1124551-supplementary-UPDATE/FigS10.pdf]
