# Supplementary material for: Preclinical Evaluation of the Association of the Cyclin-Dependent Kinase 4/6 Inhibitor, Ribociclib, and Cetuximab in Squamous Cell Carcinoma of the Head and Neck
Source: Cancers (Basel). 2021 Mar 12;13(6):1251. doi: 10.3390/cancers13061251 (PMC7998503; doi:10.3390/cancers13061251)
Supplement: Supplementary file 1 [file cancers-13-01251-s001.zip › cancers-1124551-supplementary-UPDATE/Figure S1-S9.docx]

Preclinical Evaluation of the Association of the Cyclin-
Dependent Kinase 4/6 Inhibitor, Ribociclib, and Cetuximab in Squamous Cell Carcinoma of the Head and Neck

Gabrielle van Caloen, Sandra Schmitz, Cédric van Marcke, Xavier Caignet, Antonella Mendola,
Sébastien Pyr dit Ruys, Pierre P. Roger, Didier Vertommen and Jean-Pascal Machiels


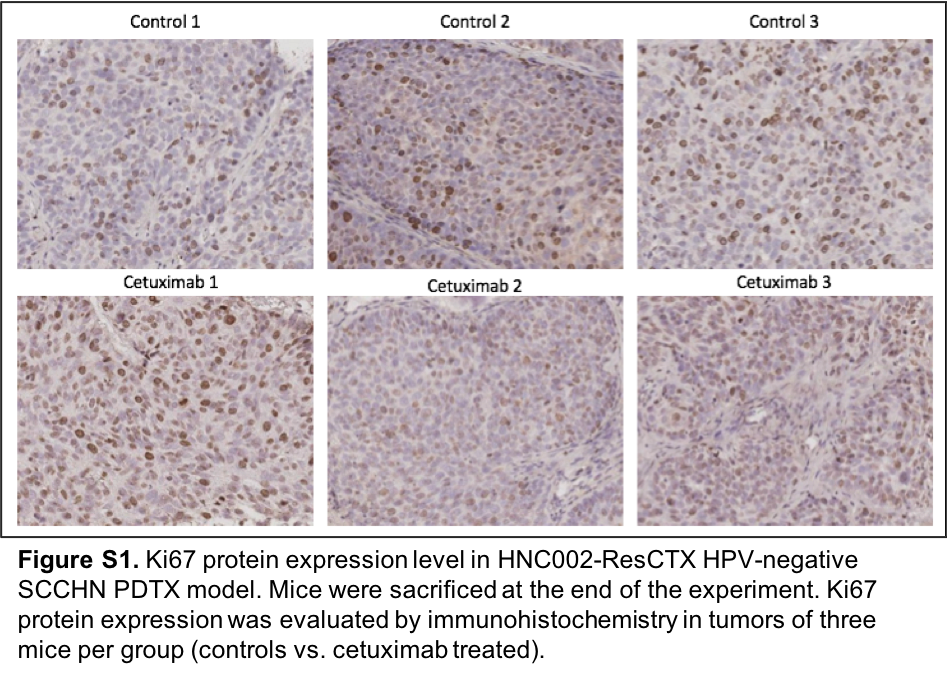


**Figure S1.** Ki67 protein expression level in HNC002-ResCTX HPV-negative SCCHN PDTX model.


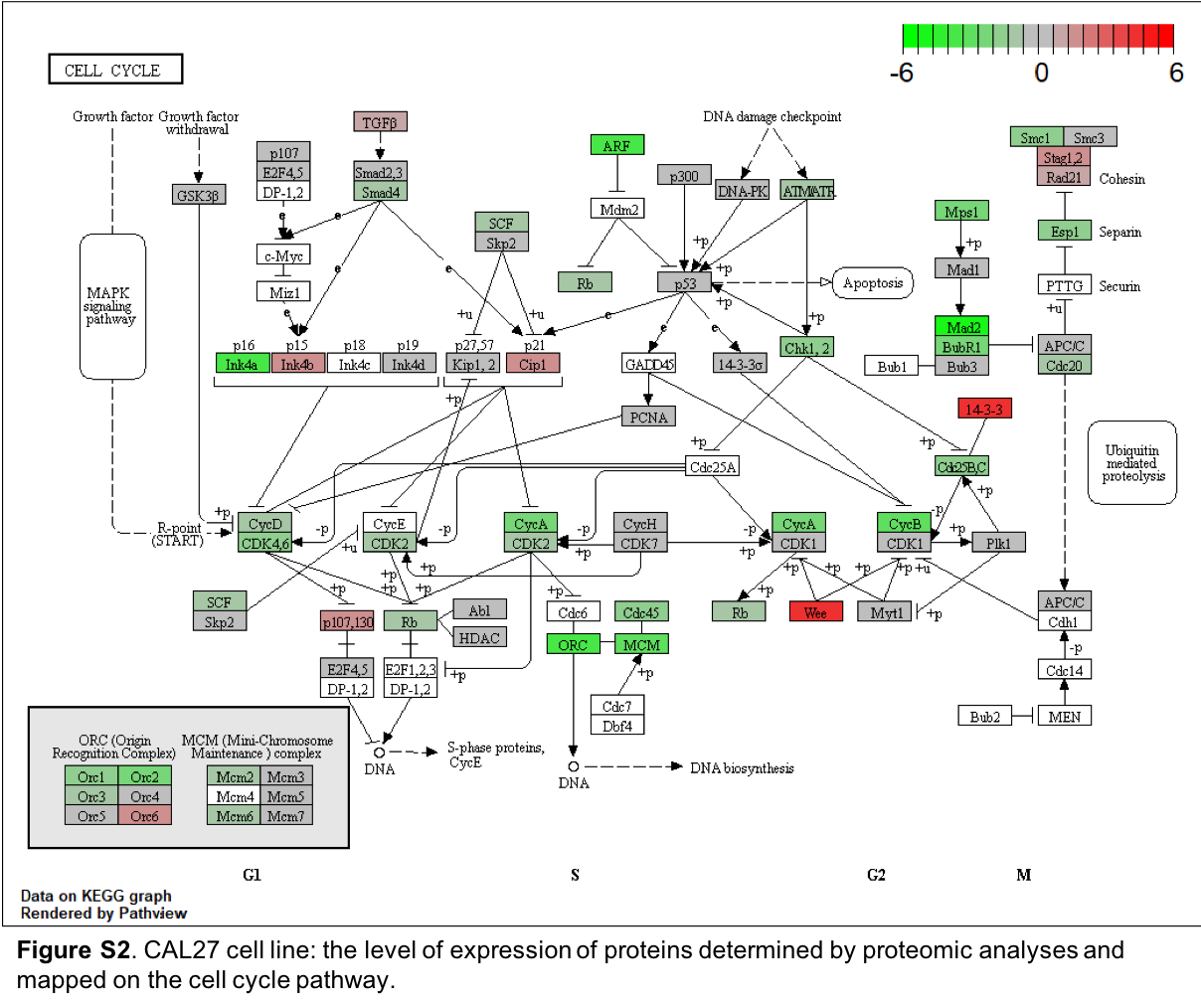


**Figure S2.** CAL27 cell line: the level of expression of proteins determined by proteomic analyses and mapped on the cell cycle pathway.


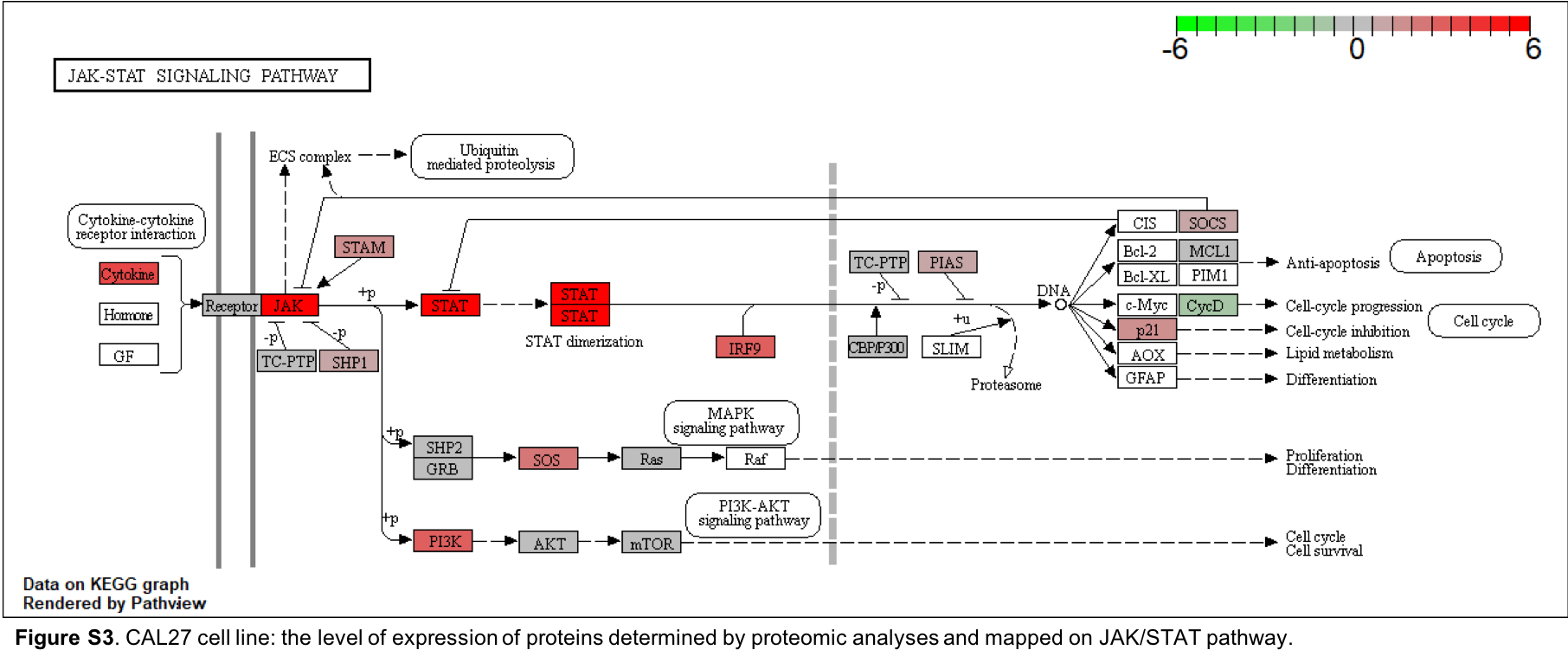


**Figure S3.** CAL27 cell line: the level of expression of proteins determined by proteomic analyses and mapped on JAK/STAT pathway.


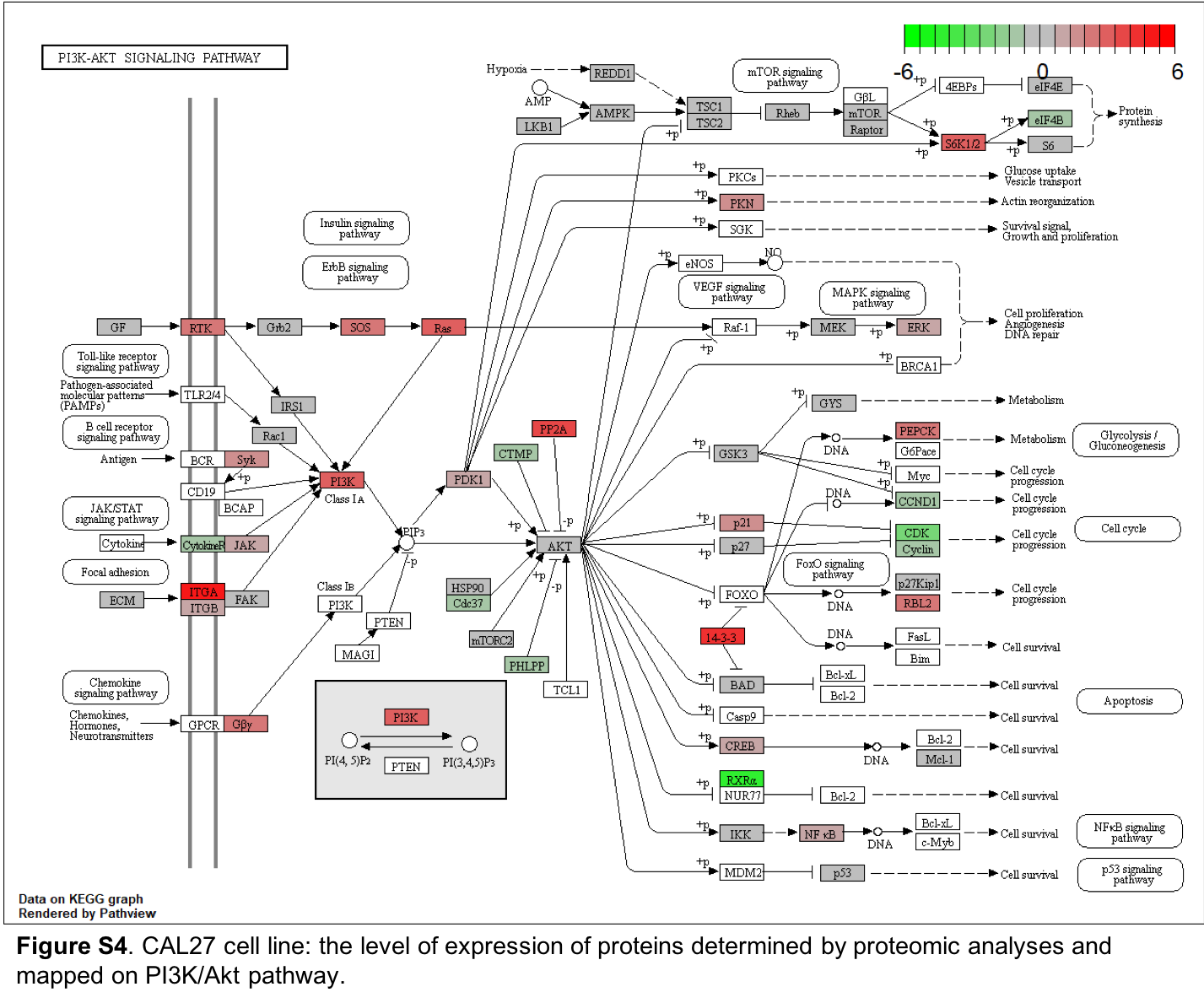


**Figure S4.** CAL27 cell line: the level of expression of proteins determined by proteomic analyses and mapped on PI3K/Akt pathway.


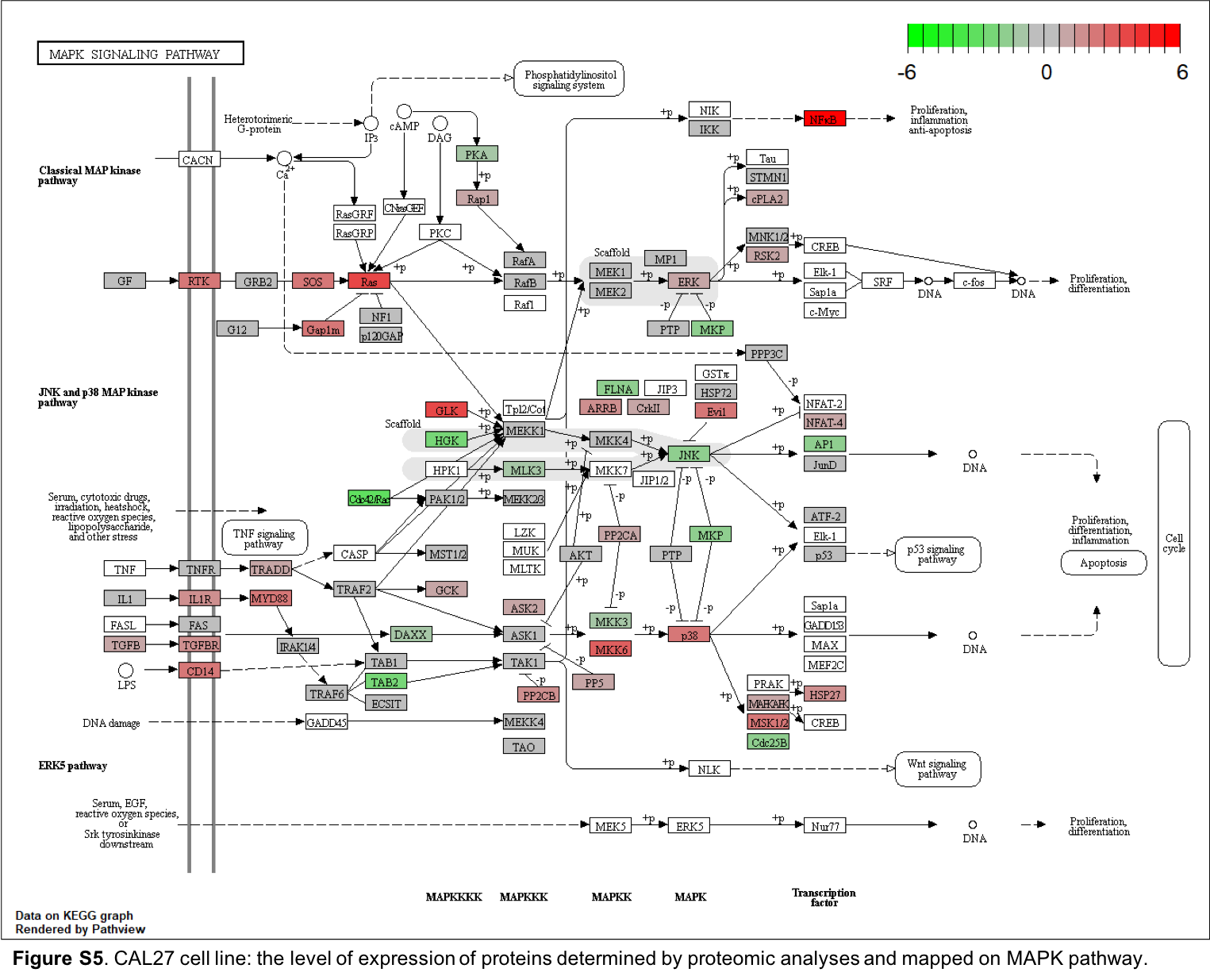


**Figure S5.** CAL27 cell line: the level of expression of proteins determined by proteomic analyses and mapped on MAPK pathway.


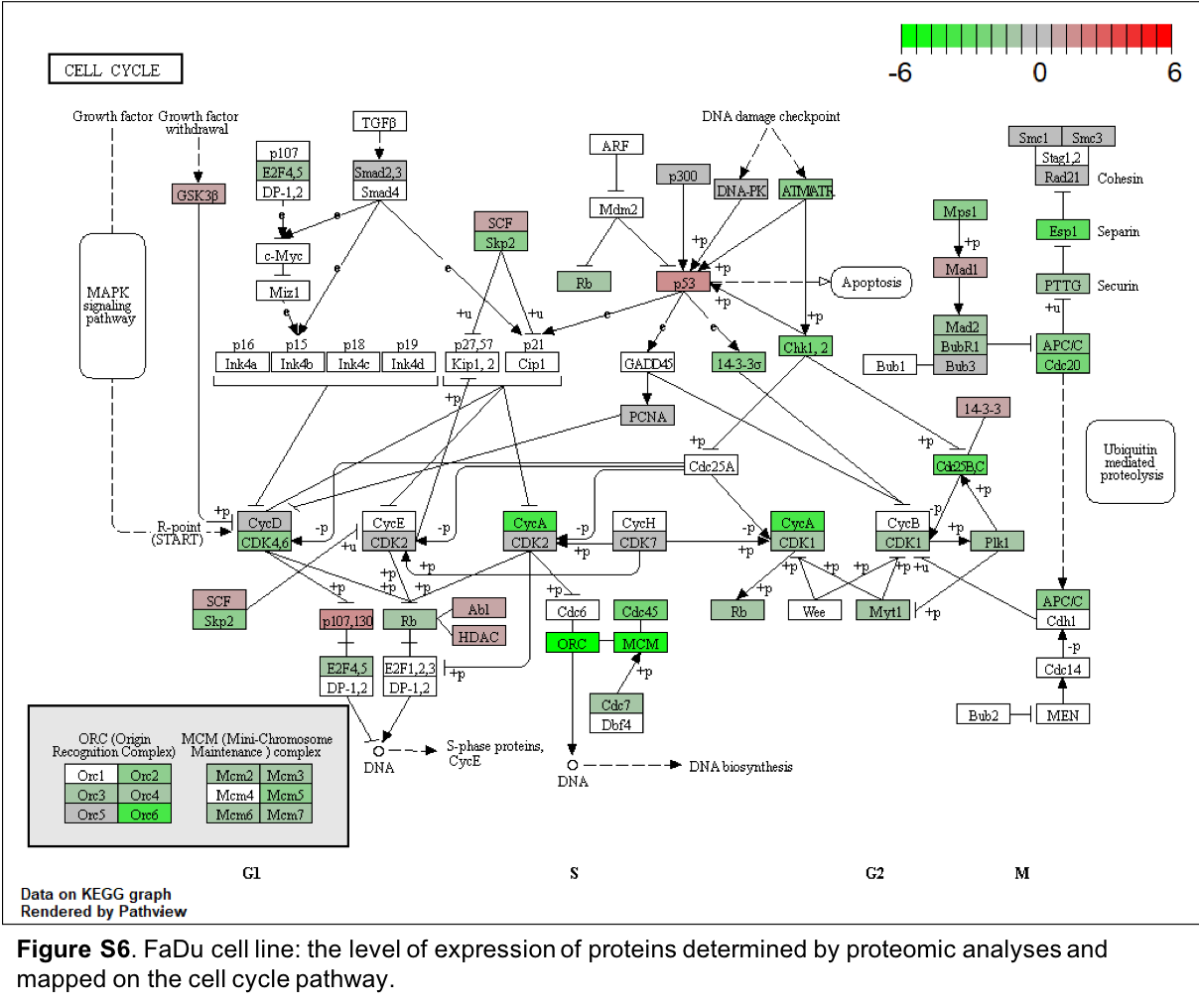


**Figure S6.** FaDu cell line: the level of expression of proteins determined by proteomic analyses and mapped on the cell cycle pathway.


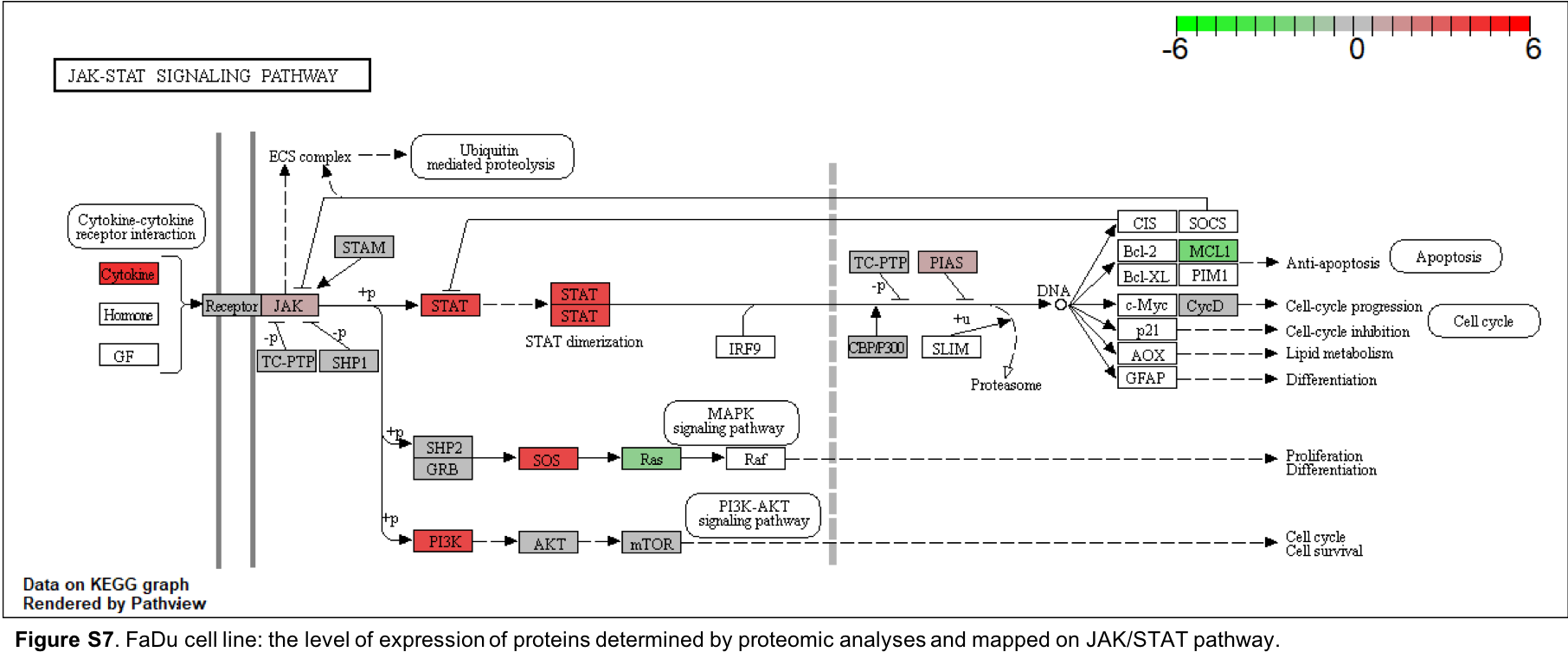


**Figure S7.** FaDu cell line: the level of expression of proteins determined by proteomic analyses and mapped on JAK/STAT pathway.


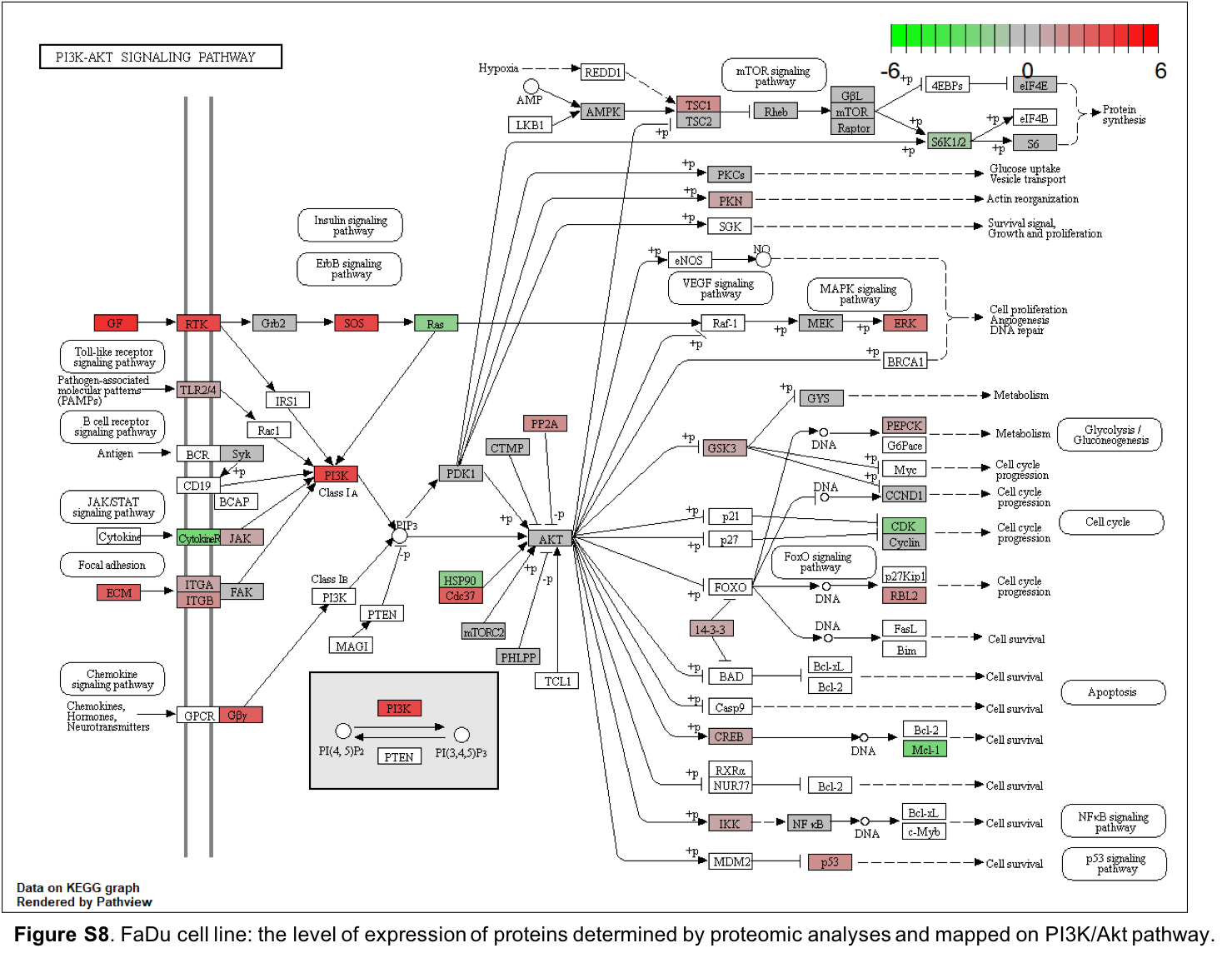


**Figure S8.** FaDu cell line: the level of expression of proteins determined by proteomic analyses and mapped on PI3K/Akt pathway.


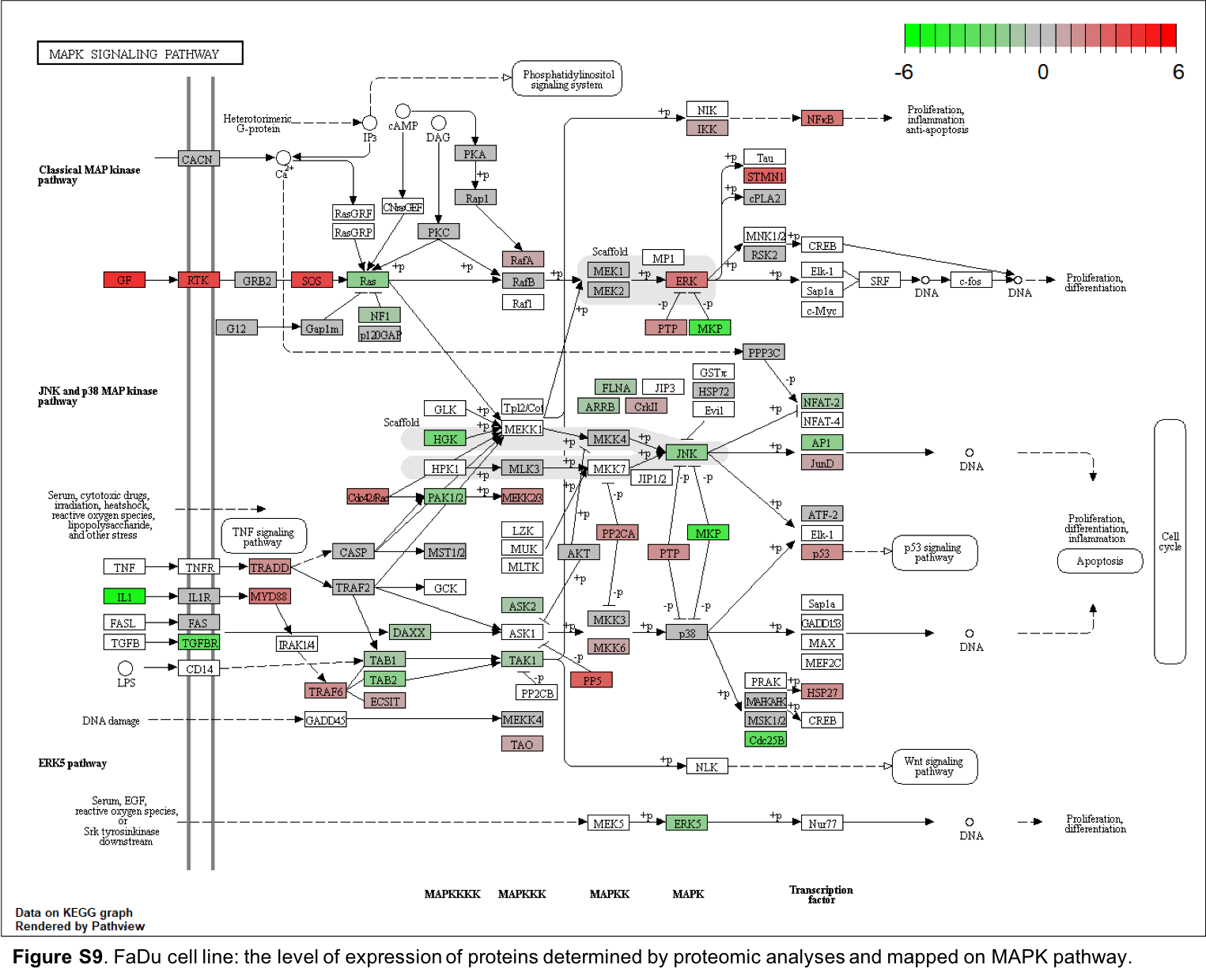


**Figure S9.** FaDu cell line: the level of expression of proteins determined by proteomic analyses and mapped on MAPK pathway.
